# Big Data and Digitalization in Dentistry: A Systematic Review of the Ethical Issues

**DOI:** 10.3390/ijerph17072495

**Published:** 2020-04-06

**Authors:** Maddalena Favaretto, David Shaw, Eva De Clercq, Tim Joda, Bernice Simone Elger

**Affiliations:** 1 Institute for Biomedical Ethics, University of Basel, 4056 Basel, Switzerland; david.shaw@unibas.ch (D.S.); eva.declercq@unibas.ch (E.D.C.); b.elger@unibas.ch (B.S.E.); 2 Department of Reconstructive Dentistry, University Center for Dental Medicine Basel, 4058 Basel, Switzerland; tim.joda@unibas.ch

**Keywords:** Big Data, digital dentistry, oral health, ethical issues

## Abstract

Big Data and Internet and Communication Technologies (ICT) are being increasingly implemented in the healthcare sector. Similarly, research in the field of dental medicine is exploring the potential beneficial uses of digital data both for dental practice and in research. As digitalization is raising numerous novel and unpredictable ethical challenges in the biomedical context, our purpose in this study is to map the debate on the currently discussed ethical issues in digital dentistry through a systematic review of the literature. Four databases (Web of Science, Pub Med, Scopus, and Cinahl) were systematically searched. The study results highlight how most of the issues discussed by the retrieved literature are in line with the ethical challenges that digital technologies are introducing in healthcare such as privacy, anonymity, security, and informed consent. In addition, image forgery aimed at scientific misconduct and insurance fraud was frequently reported, together with issues of online professionalism and commercial interests sought through digital means.

## 1. Introduction

The sophistication and increased use of Internet and Communication Technologies (ICT), the rise of Big Data and algorithmic analysis, and the origin of the Internet of Things (IOT) are a plethora of interconnected phenomena that is currently having an enormous impact on today’s society and that is affecting almost all spheres of our lives. In recent years, we have seen an exponential growth in the generation, storage, and collection of computational data and the digital revolution is transforming an increasing number of sectors in our society [1,2].

In the biomedical context, for instance, digital technologies are finding numerous novel applications to improve healthcare, cut costs for hospitals, and maximize treatment effectiveness for patients. Examples of such implementations include the development of electronic health records (EHRs) and smarter hospitals for increased workflow [3], personalized medicine and linkage of health data [4], clinical decision support for novel treatment concepts [5], and deep learning and Artificial Intelligence (AI) for diagnostic analysis [6]. In addition, the implementation of mobile technologies into the medical sector is fundamentally altering the ways in which healthcare is perceived, delivered, and consumed. Thanks to the ubiquity of smartphones and wearable technologies, mobile health (mHealth) applications are currently being explored by healthcare providers and companies for remote measurement of health and provision of healthcare services [7].

Dentistry, as a branch of medicine, has not remained unaffected by the digital revolution. The trend in digitalization has led to an increased production of computer-generated data in a growing number of dental disciplines and fields—for example, oral and maxillofacial pathology and surgery, prosthodontics and implant dentistry, and oral public health [8,9,10]. For this reason, research in the field of dental medicine is currently focusing on exploring the numerous potential beneficial applications of digital and computer-generated data both for dental practice and in research. Population-based linkage of patient-level information could expand new approaches for research such as assisting with the identification of unknown correlations of oral diseases with suspected and new contributing factors and furthering the creation of new treatment concepts [11]. AI applications could help enhance the analysis of the relationship between prevention and treatment techniques in the field of oral health [12]. Digital imaging could promote accurate tracking of the distribution and prevalence of oral diseases to improve healthcare service provisions [13]. Finally, the creation of the digital or virtual dental patient, through the application of sophisticated dental imaging techniques (such as 3D con-beam computed tomography (CBCT) and 3D printed models) could be used for precise pre-operative clinical assessment and simulation of treatment planning in dental practice [9,14]. As these technologies are still at the early phases of implementation, technical issues and disadvantages might also emerge. For instance, data collection for the implementation of Big Data applications and AI must be done systematically according to harmonized and inter-linkable data standards, otherwise issues of data managing and garbage data accumulation might arise [15]. AI for diagnostic purposes is still in the very early phases, where its accuracy is being assessed, and although they are revealing themselves to be valuable for image-based diagnoses, analysis of diverse and massive EHR data still remains challenging [16]. Finally, with regards to the simulation of a 3D virtual dental patient, dataset superimposition techniques are still experimental and none of the currently available imaging techniques are sufficient to capture the complete dataset needed to create the 3D output in a single-step procedure [9].

In the past few years, alongside the ambitious promises of digital technologies in healthcare, the research community has also highlighted many of the potential ethical issues that Big Data and ICT are raising for both patients and other members of society. In the biomedical context, data technologies have been claimed to exacerbate issues of informed consent for both patients and research participants [17,18], and to create new issues regarding privacy, confidentiality [19,20,21], data security and data protection [22], and patient anonymization [23] and discrimination [24,25,26]. In addition, recent research has also emphasized additional pressing challenges that could emerge from the inattentive use of increasingly sophisticated digital technologies, such as issues of accuracy and accountability in the use of diagnostic algorithms [27] and the exacerbation of healthcare inequalities [25].

As dentistry is also undergoing the digital path, similar ethical issues might emerge from the application of ICT and Big Data technologies. To the best of our knowledge, there is currently no systematic evaluation of the different ethical issues raised by Big Data and ICT in the field of dentistry, as most of the literature on the topic generally focuses on non-dental medicine and healthcare [28]. As timely ethical evaluation is a consistent part of appropriate health technology assessment [29] and because recent literature has focused on the ethical issues concerning health-related Big Data [28], it is of the utmost importance to map the occurrence of the ethical issues related to the application of heterogeneous digital technologies in dental medicine and to investigate if specific ethical issues for dental Big Data are emerging.

We thus performed a systematic review of the literature. The study has the following aims: (1) mapping the identified ethical issues related to the digitalization of dental medicine and the applications of Big Data and ICT in oral healthcare; (2) investigating the suggested solutions proposed by the literature; and (3) understanding if some applications and practices in digital dentistry could also help overcome some ethical issues.

## 2. Materials and Methods

We performed a systematic literature review by searching four databases: PubMed, Web of Science, Scopus, and Cinahl. The following search terms were used: “big data”, “digital data”, “data linkage”, “electronic health record *”, “EHR”, “digital *”, “artificial intelligence”, “data analytics”, “information technology”, “dentist *”, “dental *”, “oral health”, “orthodont *”, “ethic *”, and “moral *”. No restriction was placed on the type of methodology used in the paper (qualititative, qualitative, mixed methods, or theoretical). No time restriction was used. In order to enhance reproducibility of the study, we only included original research articles from peer-reviewed journals; therefore, grey literature, books (monographs and edited volumes), conference proceedings, dissertations, and posters were omitted. English was selected as it is the designated language of the highest number of peer-reviewed academic journals. The search was performed on 24 of January 2020 (see Table 1).

We followed the protocol from the Preferred Reporting Item for Systematic Reviews and Meta-Analyses (PRISMA) method [30], which resulted in 510 papers. We scanned the results for duplicates (125) and 385 papers remained. In this phase, we included all articles that focused on digitalization of dentistry or on one specific digital technology in the field of dentistry and that mentioned, enumerated, discussed, or described one or more ethical challenge related to digitalization. Papers that only described a technology from a technical point of view, that did not focus on dentistry or focused generally on medical practice, or that did not relate to the ethical challenges of digitalization were excluded. Additional papers (27) were excluded because they were book sections, posters, conference proceedings, or not in English. In total, 356 papers were excluded.

**Figure 1:**
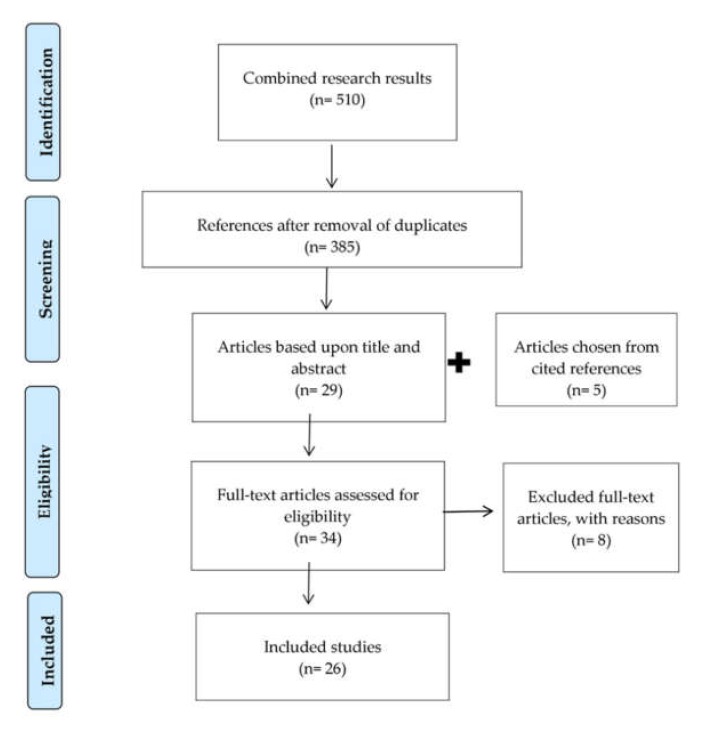
Preferred Reporting Item for Systematic Reviews and Meta-Analyses (PRISMA) flowchart.

We subsequently scanned the references of the remaining 29 articles to identify additional relevant studies. We added five papers through this process. The final sample included 34 articles. During the next phase, the first author read the full texts in their length. After thorough evaluation, eight articles were excluded for the following reasons: (1) they did not discuss or mention any ethical issue related to the technology discussed in the study; and (2) they did not refer to any digital implementation in dentistry (see Figure 1).

The subsequent phase of the study involved the analysis of the remaining 26 articles. Regarding data analysis, we carried out a narrative synthesis of included publications [31]. Therefore, we extracted the following information relevant to the aim of the present study and to the research question from the papers: year and country of publication; methodology; type of technology or digital application discussed; field of application of the article; ethical issues that emerge from the use of the technology; technical issues that might exacerbate the ethical issues discussed; suggested potential solutions to the issue(s); and ethical issues that the technology could help overcome.

## 3. Results

Among the 26 papers included in our analysis, 22 were theoretical papers that critically discussed the impact of digitalization in the field of dentistry or that discussed a specific technology highlighting its promises and some of its ethical challenges. Among the remaining papers, three applied empirical methods and one was a feasibility study. The majority of papers (n = 20) were published after 2010, five were published between 2008 and 2010, and one of them was from 1996. Half of the articles (n = 13) were from the United States, five came from the United Kingdom, and four from India. The remaining ones came from Belgium, Brazil, Germany, and South Africa. Regarding the type of technological application they discussed, almost one-third of the papers (n = 8) analyzed digital photography, radiology and computed imaging; six papers discussed the impact of digital communication and social media in dentistry; three articles focused on electronic health records (EHRs) and patient records; another three discussed the promises and challenges of mobile health and teledentistry; and an additional three records focused on data linkage and personalized medicine. In addition, two papers broadly discussed the challenges and promises of ICT and digital implementations in dentistry, while one paper focused on search engine optimizations in dental practices. Finally, concerning the field of application of the different papers, 10 articles discussed the ethical issues of digitalization regarding dental practice, nine discussed digitalization and digital application for dentistry without a specific focus, five focused on education and dental school, and two discussed applications in research (see Table 2).

### 3.1. Implementation of Digital Technologies in Dentistry

Two papers generally discussed the ethical implications that ICT and digitalization are introducing in dentistry [32,33]. According to Gross et al. [32], digitalization of dentistry is influencing the patient doctor relationship as the integration of digital technologies could distract attention away from the patient during the visit. Issues of data literacy can arise for both the dentist—who will need to constantly be updated on the latest technologies—and the patient—who will need to understand how new technologies work, possibly disfavoring people with poor computer literacy such as the elderly. The application of AI for diagnostic purposes could create issues of responsibility and accountability. A shift might occur towards overtreatment of the patient owing to increased demand for the use of digitized systems. In addition, the constant use, refurbishment, and replacement of increasingly new technology leaves a remarkable digital footprint and aggravates digital pollution. Finally, digital technologies create issues of data security, data falsification, and privacy issues regarding identifiable patient information [33].

### 3.2. Big Data and Data Analytics

Nine papers discussed the increased employment of Big Data and data analytics in dentistry related to different applications such as data linkage [34], data analytics in dental schools [35], personalized medicine [36], EHRs [37,38,39], and mHealth and teledentistry [40,41,42].

#### 3.2.1. Electronic Health Records (EHRs)

Three papers focused on the implementation of EHRs both in private practices and in dental education [37,38,39]. Ethical issues that arise from this technology are data security, as sensitive patient information could be more easily accessed by unauthorized third parties, resulting in a breach of patient privacy and confidentiality [38,43].

In addition, Cederberg and Valenza [38] argue that the use of digital records might compromise the doctor patient relationship in the future, as easy access to all relevant information through digital means and forced focus on the computer screen could accustom students to becoming more detached from patients.

Suggested solutions for privacy and security issues related to EHR are as follows: (a) the implementation of a three-zone confidentiality model of medical information for databases both linked (networked) and non-linked (network), where different levels of access and security are put in place for different areas—from a more secured inner area that holds the highest sensitive information about the patients (e.g., HIV status and psychiatric care) to an outer, less secured area containing generally publicly available information [37].

#### 3.2.2. mHealth and Teledentistry

Ethical concerns related to mHealth and teledentisry—that is, the use of information technologies and telecommunications to provide remotely dental care, education and raise oral health awareness—were raised by three articles [40,41,42]. As for other Big Data technologies, issues of data security and patient anonymity [40,41] and confidentiality [42] were the most mentioned, as networked transfer through unsecure means could enable unwarranted third parties to obtain easier access to sensitive patient data.

mHealth might also have an impact on consent both for the patient who might not have been appropriately informed about all of the risks that teledentistry implies [42] and for non-consenting bystanders, whose data might be collected by the device the patient is using [41].

Furthermore, Cvkrel [41] argued that first, mHealth creates additional vulnerability as smartphones gather additional data that are usually not collected by healthcare practitioners (e.g., fitness data, sleep patterns), and, as it is an object of everyday use, it might be easily accessible to unauthorized people. Second, easy access through the smartphone to raw data including data related to dental care could be counterproductive and harmful for patients who might self-adjust the prescription given by the practitioner.

Among the suggested solutions are the following: (a) the establishment of secured networking communication such as the development of state-of-the-art firewalls and antiviruses to mitigate security concerns in telecommunications [40]; (b) the formulation of high quality consent processes that appropriately make the user aware of the risks and all relative factors [41]; and (c) the implementation of information and education about the specific issues that such technology raises for dentists who want to employ teledentistry in their practice.

#### 3.2.3. Personalized Medicine and Data Linkage

In the context of data linkage in dental practices, personalized medicine, and dental schools, the analyzed articles reported how consent issues might arise concerning data usage when the student or the patient cannot be completely informed about the ways in which the collected data is used [35]. Data anonymization [34] and patient confidentiality [36] were again both mentioned as issues of data linkage. Finally, Eng et al. [36] highlighted how discrimination based on higher risk for specific diseases might appear from the linkage of different databases in personalized medicine.

In order to overcome these issues, Eng et al. [36] suggested to develop protective measures at both at a legal and a clinical level to ensure patient data confidentiality and security.

### 3.3. Digital Communication and Social Media in Dentistry

Seven papers discussed the impact that the employment of digital communication and social media could have upon dental practices and the dentist–patient relationship [44,45,46,47,48,49,50].

According to the retrieved studies, one of the main issues is the possibility that commercial values might creep into the management of private practices’ websites and official social media pages [44]. For instance, digital media broadcasts might deliver a distorted image of the practice, resulting in misleading or dishonest advertisement of state-of-the-art dental technologies or dental practices, thus exercising an undue influence on patients [47,49]. In addition, Swirsky [50] also raised a concern regarding unethical search engine optimization, an aggressive marketing technique aimed at making your own website appear before others in popular search engines. This practice creates conflict of interest between the dental profession and the patient/public.

Furthermore, the introduction of digital communication in dental practices has heavy effects on the dentist–patient relationship. Neville and Waylen [45] indicate how the use of social media pages is blurring the personal and professional divide. Via social media, patients might have access to information about their dental providers that could compromise the doctor–patient relationship and create issues of trust between the two parties. For instance, shared posts and messages of doctors might be misinterpreted by the users (patients) and be considered unprofessional. Likewise, privacy issues might occur in the case where a dentist visits the personal social media page of their patient and uncovers information that the patient did not want to share with them [46,48]. In addition, doctor–patient confidentiality could be breached by dentists both willingly and inadvertently, if information about a patient is disclosed online, such as identifiable patient photographs or sensitive treatment details [47,49].

Suggested practices to avoid such issues are the development of adequate social media policies for the use of social media in dental practices and increased education for dental practitioners regarding online professionalism in social media—such as awareness of the ethical issues and of the rules of conduct to be used while using social media [48,49].

### 3.4. Digital Photography and Radiography

The technology discussed by eight of the collected papers was digital photography and digital radiography [51,52,53,54,55,56,57,58]. Among them, four articles [51,53,55,56] highlighted that image modification, made easier by digitalization of both dental photography and radiography, could result in misconduct in science and fraudulent use of modified pictures. Practitioners could be tempted to modify radiographs to deceive insurance companies [51] and researchers might do the same to falsify the results of their research [55].

Three papers correlated the ethical issues of digital imagery to digital sharing and storage of images [52,57,58]. For instance, issues of security of data and patient privacy and confidentiality might arise owing to inattentive storage of images (if digital photographs are stored for too long on an SD-card or if images are shared via electronic means such as using emails and smartphones or networking apps as Whatsapp) [52]. In addition, Stieber et al. [57] indicate how even patient autonomy and consent might be breached if the images are used in an unauthorized manner, such as posting them on a public forum.

Finally, one paper that discussed the ethical issues of digital dental imaging focused on a particular diagnostic technology: cone beam computed tomography (CBCT) [54]. Highlighted issues related to this particular technology are related to its routine use potentially causing harm to patients, especially children and adolescents, owing to the excessive exposure to radiation and consent if patients are not appropriately informed about the health risks they are exposed to when undergoing this diagnostic exam.

Some papers also highlighted some potential solutions. Regarding image modification, the application of state-of-the-art anti-forgery techniques was suggested [51], as well as the development of appropriate guidelines to set an acceptable standard for image modification in dentistry [53]. As for image sharing issues, Stieber et al. [57] suggested the implementation of a privacy compliant framework, where informed consent is enhanced in order to give patients more control over how their images are used, while Indu et al. [52] proposed the use of only custom apps built exclusively for medical data sharing.

### 3.5. Digital Dentistry Might Solve Ethical Issues

Finally, almost one-third of the papers discussed not only ethical issues, but also mentioned how some of these technologies could be of assistance to solve ethical issues in dentistry and oral health. For instance, the application of digital technologies could result in empowerment of patients and democratization of oral health knowledge owing to increased and widespread information that could be easily retrieved on the Internet [32]. mHealth and teledentistry were argued to be powerful tools to (a) fight known inequalities in healthcare and provide better treatment and patient care in vulnerable populations thanks to the increased saturation of mobile phones and communication technologies that will allow them easier access to health information and remote treatment [41]; (b) overcome cultural and geographic barriers in oral health [40]; and (c) help eliminate the disparities in oral health care between rural and urban communities [42]. Provision of information about health care prevention and oral health issues through social media could positively influence and promote oral healthcare [46,49]. The implementation of research through correlation and data linkage between birth cohorts in the United Kingdom and oral health habits could ameliorate public oral health issues such as caries prevention for children and adolescents [34]. Finally, digital forensics, that is, the digital analysis of images, could help with the recognition of scientific misconduct in dental research [55].

## 4. Discussion

The analyzed literature raised a plethora of intertwined ethical issues across different technologies and practices in dentistry. Numerous issues are in line with the commonly mentioned ethical challenges that digital technologies are introducing in healthcare—privacy anonymity, security, and so on. On the other hand, additional aspects emerged for dental medicine—such as commercialization and image forgery—that are usually less associated with digitalization of healthcare and Big Data [28].

The most frequently mentioned ethical issues related to the increased digitalization of dentistry are those related to patient privacy, which is often associated with anonymization and confidentiality. This is in line with a study by Mittelstadt and Floridi [28] that highlighted how this cluster of issues related to patient privacy is the one that is most correlated by scholarly research with Big Data technologies such as data analytics, IOT, and social media use. In the era of digitalization, with increased implementation of EHRs and digital data management, issues of privacy become among the most paramount, notably also in dentistry, on account of the opportunities for patient treatment development and research offered by data linkage. Important ethical issues could be overlooked if it is assumed that dental health data are less sensitive than, for example, mental health or stigmatizing infectious disease data. On the contrary, dental health data are sensitive for a number of specific reasons. For example, economic or marketing discrimination, that is, inequality in pricing and offers that are given to costumers based on profiling, such as insurance or housing [59], or discrimination based on health data and health prediction [60], are practices that are creeping out of the exploitation of digital records and might be exacerbated by the analysis of dental records and the use of mHealth in dentistry.

Informed consent was another issue that was often mentioned by the selected papers, although surprisingly not in relationship to the reuse of EHR data. From an ethical and legal point of view, consent needs to be specific concerning three different activities: use for clinical care; clinical trials, where new Big Data technologies are used in dental patients; and secondary use of data for research or other purposes (such as marketing). For use in the clinical setting, issues of informed consent are not so prominent as the EHR would function as a substitute for a paper patient chart, leaving more concerns in the area of data security and patient privacy. However, as Big Data applications for secondary use of EHR data are becoming an increasingly implemented research practice and issues of consent for EHR and Big Data are quite often discussed for the biomedical context [28], more research should be spent in this area for the dental field. In fact, only three retrieved papers focused on EHR—they mostly targeted clinical care, and two of them were from before 2010, which may explain why they did not consider the implications of Big Data and secondary use of data from health records that are currently causing dilemmas of consent from both an ethical and a regulatory point of view [17,61]. Consent was also briefly mentioned by the retrieved papers in relation to data linkage and personalized medicine, but overall, the literature has not sufficiently analyzed the issue data linkage and secondary use of data for dentistry. In fact, electronic dental records increasingly include sensitive and complementary data about the patient, such as automatic tooth charting, general patient health information, development of treatment plans, radiographic captures of the mouth, and intraoral photography [43], which could be linked and analyzed for research and app development purposes without obtaining the appropriate patient’s approval. Cvrkel [41], in the context of mHealth, suggested deflecting the discussion from privacy concerns to the development of high-quality consent practices for both clinical as well as secondary research use. On the basis of a recent study by Valenza et al. [62], which assessed the benefits of “Smart consent” strategies that take into account patients’ preferences and desires regarding both treatment and the use of their dental data, we argue that the implementation of better consent policies and strategies could also be beneficial to electronic dental records in order to face not only privacy issues related to clinical care, but also issues of consent related to secondary use of data.

As might be expected, considerable space was given to digital photography and radiology in dentistry. Ethical issues were raised in two directions. First, concerns of patient privacy and anonymity and of data security were highlighted in relation to the storage and sharing of digital images [52,57,58]. These issues are of a comparable nature to those enumerated for EHR, mHealth, and teledentistry, which principally have to do with possible access to sensitive patient information by unwarranted parties and interception of digital communications. Interestingly, substantial weight was given to the topic of image forgery. According to the literature, image modification for fraudulent purposes such as insurance fraud and scientific misconduct is described as an expanding practice within dentistry [55,56]. The main problem is that the introduction of digital imagery in our society has exponentially increased the ease with which digital photographs can be manipulated and changed, both in the early and late stages of image production, to a point where essential information about the subject of the image might be falsified [63]. As a consequence, numerous scholars who focused on the epistemic status of photographs and digital imaging have tried to analyze the challenges that digital imaging poses to the epistemic consistency of images [63,64,65]. The question is, in our opinion, whether in the case of image modification in dentistry, a well-defined line can be settled on acceptable modifications that prevent misinterpretation or misreading by the observer, and modifications that would let the image fall in the category of image forgery. Following clear guidelines on the ethics of image modification [66] could assist practitioners in making the right choices, but might not be enough. Well-intentioned image modification, such as changing the background, modifying light sources, over and under exposure, cropping, color modification, and so on might unintentionally alter the epistemic consistency of an image, as the limit of acceptable alterations that digital images can endure, while maintaining their epistemic value is vague and undetermined [63].

Another interesting finding of this study is that numerous articles—almost one-third of the total and all theoretical papers—rather than expanding on the ethical issues that derive from the application of a medical/dental digital technology, focused on how digital communication could have an impact on the practice of dental care itself and on the doctor–dentist relationship. Some of the retrieved papers [44,45,46,47,48,49], in fact, highlighted how the inappropriate use of social media by dentists could compromise trust between dental practitioners and patients either owing to leakage of confidential information about patients, such as treatment outcomes or identifiable pictures, or displays of inappropriate behavior on their private social media pages. As the use of social media is permeating our everyday life, blurring the line between private and public, social media and online professionalism are topics that have been increasingly addressed in other areas of healthcare as well [67,68]. The ethical challenge here seems to be twofold. First, education regarding the professional use of social media for dental practitioners could be enhanced by the implementation of rules and social media policies that clearly state the “dos-and-don’ts” of managing a social media page, such as the following: do not post identifiable pictures of patients without their consent; do not discuss patient treatment on the page, and so on [48]. However, if a breach of confidentiality should occur through inattentiveness, the reach of the leaked information would be greater than in face to face exchanges, expanding exponentially the scale of the mistake [67]. Second, it becomes more challenging to implement strategies to appropriately educate dental practitioners about their private social media behavior. It has been argued by Greysen et al. [67] that some online content that might be flagged as unprofessional—such as posts concerning off-duty drinking and intoxication or the advertisement of radical political ideals that might question their professionalism—do not clearly violate any existing principle of medical professionalism, as they are done in the private sphere. In addition, even the interactions that a health practitioner might have with the private social media page of a patient become an intricate matter that might raise ethical dilemmas. By only accessing the page of their patient, the doctor could access private information such as their marital status, sexual orientation, or political orientation that might have an impact, either conscious or unconscious, on the practitioner’s personal perception of the patient [69]. Things become even more complicated if the healthcare professional retrieves posts or photos on social media sites that depict patients participating in risk-taking or health-averse behaviors [67]. All of this information might create a fracture in the patient–doctor relationship, as implicit bias and conflict of interests might prevent medical practitioners from providing the patient with the best care [69,70].

In addition, another interesting challenge raised by almost all of the papers that discussed digital communication in dentistry was the issues of commercialization and conflict of interest that interfere with patient care. A strong focus of some of the papers was on the possible exertion of undue influence on the patient by producing misleading advertisement for private practices and state-of-the-art dental procedures. As Chambers et al. [44] argue, the dentist–patient relationship should never shift to one of customer–provider, and commercial interests should always be in a subordinate position to that of oral health, as the well-being of the patient should always come first. In addition, according to the American Dentist Associations’ (ADA) Code of Conduct: “dentists who, in the regular conduct of their practices, engage in or employ auxiliaries in the marketing or sale of products or procedures to their patients must take care not to exploit the trust inherent in the dentist–patient relationship for their own financial gain […] and no dentist shall advertise or solicit patients in any form of communication in a manner that is false or misleading in any material respect” [71].

Doing so would negate the patient’s right to self-determination and accurate information [50]. As additional technological developments are being increasingly introduced in dental practices, it is of the utmost importance that strong measures are taken to limit commercial interests for dental practice.

In addition, while a substantial number of papers focused on digital photography and radiography, as well as the impact of digital communication for dental practice, this systematic review highlighted some gaps regarding some of the applications that data technologies have in dentistry and the possible ethical issues that might emerge as a consequence. For instance, the implementation of AI applications for diagnostic purposes in dentistry [12] or the sophistication of 3D imaging technologies for pre-operative clinical assessment [9] were not discussed in the retrieved literature. In addition, very few of the retrieved papers focused on the increased application of Big Data analytics and data linkage of health-related data. Shetty et al. [72] highlighted how the debate on digital dentistry is reflective of the traditional dental delivery model and usually focuses on micro trends in technology development such as technology-assisted services (e.g. computer-aided design/computer-aided manufacturing (CAD/CAM)), digital radiography, and electronic patient records. However, trends in the implementations of Big Data technologies such as mHealth, social media, AI, and the like are transforming oral healthcare through social and technical influences from outside the dental profession, as has been seen in relation to the social media use by dental providers. In addition, it has recently been argued that current literature on the topic of digital dentistry has a tendency to focus on its beneficial potentials or on the technical challenges of the discussed technology without appropriately addressing the ethical issues that these technologies might raise [32]. Also, our review indicates that, while a theoretical discussion on this topic is emerging, empirical studies on the ethical issues of digital implementations in dentistry are largely lacking. As a consequence, owing to the sensitive nature of data included in electronic dental records, the specific digital implementations in dental practice and research, and the gaps in the literature regarding the ethical analysis of some dental applications, it is of the outmost importance to conduct additional research, and especially more evidence-based studies, on the possible specific ethical issues related to the field of digital dentistry in order to appropriately understand and confront these issues.

Finally, only a few papers mentioned ethical issues that could be solved by digital dentistry. In addition to those mentioned in Section 3.5, there are two other contenders for useful applications of Big Data research. It has historically been very difficult to conduct epidemiological research on the relationship (if any) between the public health measure of adding fluoride to water supplies and the incidence of dental fluorosis in children owing to the very high number of variables and confounders involved in such research. Big Data analytics could make sense of this difficult area of research, helping to address the public health ethics of water fluoridation [73]. Similarly, antibiotic prophylaxis before dental treatment in patients who have undergone heart surgery remains a contentious area, with dentists tending to recommend against it despite heart surgeons supporting the prescription of antibiotics [74]. Big Data research could help to shed some light on this difficult ethical dilemma.

## 5. Conclusions

Our study highlighted how most of the issues presented for digital dental technologies such as electronic dental records, mHealth, and teledentistry, as well as developments in personalized medicine, are in line with those mostly discussed in the debate regarding the application of ICT in healthcare, namely, patient privacy, confidentiality and anonymity, data security, and informed consent. In addition to those issues, image forgery aimed at scientific misconduct and insurance fraud was frequently reported in the literature. Moreover, the present review identified how major concerns in the field of dentistry are related to the impact that an improper use of ICT could have on the dental practice and the doctor–patient relationship. In this context, issues of online professionalism were raised together with issues of aggressive or misleading social media or web. Finally, additional research should be conducted to properly assess the ethical issues that might emerge from the routine applications of increasingly novel technologies.

## Figures and Tables

**Table 1:** Search terms.

No.	Match Search Terms	Pub Med	Web of Science	Scopus	Cinahl
1	(“big data” OR “digital data” OR “data linkage” OR “electronic health record*” OR “EHR” OR “digital*” OR “artificial intelligence” OR “data analytics” OR “information technology”)	251,004	4,682,526	1,750,766	67,116
2	(“dentist*” OR “dental *” OR “oral health” OR “orthodont*”)	827,547	1,409,796	613,348	158,231
3	(“ethic *” OR “moral*”)	334,537	582,299	528,738	98,246
4	1 AND 2 AND 3	190	186	71	63

**Table 2:** Retrieved papers. EHR, electronic health record; mHealth, mobile health; CBCT, con-beam computed tomography; ICT, internet and communication technologies.

Author, Year, Country	Design	Participants	Technology Discussed	Field of Application	Ethical Issues
Boden (2008), USA	Theoretical		Digital transfer of patient records	Dental practice	Justice and autonomy- high charges for the patient prevent beneficial use of records for future patient treatment
Calberson et al. (2008), Belgium	Theoretical		Digital radiography	General	Fraudulent use of radiographs
Cederberg and Valenza (2012), USA	Theoretical		EHR (in dental schools)	Dental school	Justice, patient privacy and security, shift in doctor patient relationship, misconduct from students
Chambers (2012), USA	Theoretical		Digital Communication	Dental practice	Shift in doctor patient relationship, patient privacy and security, professionalism
Cvrker (2018), USA	Theoretical		mHealth	General	Patient access, data ownership, patient privacy and security, bystanders
da Costa et al. (2012), Brazil	Theoretical		Teleorthodontics	General	Patient privacy and security
Day et al. (2018), UK	Feasibility Study	Birth cohort in the United Kingdom	Data linkage	Research	Anonymization, data ownership
Eng et al. (2012), USA	Theoretical		Personalized dentistry	General	Discrimination, confidentiality
Gross et al. (2019), Germany	Theoretical		Digitalization in dentistry	General	Shift in doctor patient relationship, data literacy, responsibility and accountability for AI, digital footprint
Indu et al. (2015), India	Empirical	A sample of postgraduate students and teaching faculties of oral pathology in India	Digital photography	General	Anonymity and security
Jampani et al (2011), India	Theoretical		Teledentistry	General	Confidentiality, patient privacy and security, consent
Kapoor (2015), India	Empirical		Digital photography and radiology	General	Fraudulent use of radiographs/photographs, scientific misconduct
Khelemsky (2011), USA	Theoretical		CBCT	Dental practice	Harm to patient, consent
Knott (2013), UK	Theoretical		ICT	Dental practice	Anonymity, data security, patient privacy
Luther (2010), UK	Theoretical		Digital forensics	Research	Fraudulent use of images, scientific misconduct,
Neville and Waylen (2015), UK	Theoretical		Social Media	Dental practice	Shift in doctor patient relationship, patient Confidentiality, privacy, anonymity
Oakley and Spallek (2012), USA	Theoretical		Social Media	Dental School	Shift in doctor patient relationship, patient privacy and confidentiality, miscommunication, boundary violation
Peltier and Curley (2013), USA	Theoretical		Social Media	Dental practice	Dishonest/unlawful advertising, patient confidentiality
Rao et al. (2010), India	Empirical	A sample of randomly selected clinicians in India	Digital photography	General	Fraudulent use of photographs, scientific misconduct
Spallek er al. (2015), USA	Theoretical		Social Media	Dental School	Shift in doctor patient relationship, patient privacy and confidentiality, miscommunication, boundary violation
Stieber et al. (2015), USA	Theoretical		Electronic media and digital photography	Dental School	Patient privacy and confidentiality, autonomy and consent
Swirsky at al. (2018), USA	Theoretical		Search engine optimization	Dental practice	Beneficence, autonomy, consent, conflict of interest and undue influence
Sykes et al (2017), South Africa	Theoretical		Social Media	Dental practice	Patient privacy, anonymity, confidentiality and consent, professionalism, shift in patient doctor relationship, misleading advertisement
Szekely et al. (1996), USA	Theoretical		EHR	Dental practice	Patient privacy and confidentiality, security
Wenworth (2010), USA	Theoretical		Digital Radiography	Dental practice	Patient privacy and confidentiality, misleading advertisement
Zijlstra-Shaw and Stokes (2018), UK	Theoretical		Big Data analytics (in dental education)	Dental school	Consent and data ownership
